# Cerebral Blood Flow Response to Simulated Hypovolemia in Essential Hypertension: A Magnetic Resonance Imaging Study

**DOI:** 10.1161/HYPERTENSIONAHA.119.13229

**Published:** 2019-10-28

**Authors:** Sandra Neumann, Amy E. Burchell, Jonathan C.L. Rodrigues, Christopher B. Lawton, Daniel Burden, Melissa Underhill, Matthew D. Kobetić, Zoe H. Adams, Jonathan C.W. Brooks, Angus K. Nightingale, Julian F. R. Paton, Mark C.K. Hamilton, Emma C. Hart

**Affiliations:** 1 From the Faculty of Life Sciences, School of Physiology, Pharmacology and Neuroscience (S.N., Z.H.A., J.B., A.K.N., J.P., E.C.H.), University of Bristol, United Kingdom; 2 Faculty of Health Sciences, Bristol Medical School (S.N., M.K.), University of Bristol, United Kingdom; 3 University Hospitals Bristol NHS Foundation Trust, United Kingdom (A.E.B., J.R., C.B.L., D.B., M.U., A.K.N., M.H.); 4 Department of Radiology, Royal United Hospitals Bath NHS Foundation Trust, United Kingdom (J.R.); 5 Department of Physiology, Faculty of Medical and Health Sciences, University of Auckland, New Zealand (J.P.).

**Keywords:** brain blood flow, cerebral blood flow, hypertension, LBNP, lower body negative pressure

## Abstract

Hypertension is associated with raised cerebral vascular resistance and cerebrovascular remodeling. It is currently unclear whether the cerebral circulation can maintain cerebral blood flow (CBF) during reductions in cardiac output (CO) in hypertensive patients thereby avoiding hypoperfusion of the brain. We hypothesized that hypertension would impair the ability to effectively regulate CBF during simulated hypovolemia. In the present study, 39 participants (13 normotensive, 13 controlled, and 13 uncontrolled hypertensives; mean age±SD, 55±10 years) underwent lower body negative pressure (LBNP) at −20, −40, and −50 mmHg to decrease central blood volume. Phase-contrast MR angiography was used to measure flow in the basilar and internal carotid arteries, as well as the ascending aorta. CBF and CO decreased during LBNP (*P*<0.0001). Heart rate increased during LBNP, reaching significance at −50 mmHg (*P*<0.0001). There was no change in mean arterial pressure during LBNP (*P*=0.3). All participants showed similar reductions in CBF (*P*=0.3, between groups) and CO (*P*=0.7, between groups) during LBNP. There was no difference in resting CBF between the groups (*P*=0.36). In summary, during reductions in CO induced by hypovolemic stress, mean arterial pressure is maintained but CBF declines indicating that CBF is dependent on CO in middle-aged normotensive and hypertensive volunteers. Hypertension is not associated with impairments in the CBF response to reduced CO.

## Abstract 2

Supplemental Digital Content is available in the text.

Hypertension is associated with cerebrovascular remodeling,^1^ a reduction in resting cerebral blood flow (CBF), and higher cerebrovascular resistance relative to healthy normotensive individuals.^2–4^ Impairment in the ability to regulate CBF carries increased risk of cerebrovascular events and dementia.^5–7^

In a large-scale longitudinal study in 575 patients with manifest arterial disease, Muller et al^3^ found the largest reduction in CBF between the baseline visit and 4-year follow-up in hypertensive patients who had a reduction in blood pressure (BP) and in patients whose BP remained high at the 4 years of follow-up. A reduction in CBF has also been observed with antihypertensive treatment,^8^ and lower CBF has been measured in treated controlled versus untreated hypertensive patients.^4^ However, the effect of antihypertensive medication on CBF control is poorly understood.^9^

In response to a BP challenge such as lower body negative pressure (LBNP), which simulates hypovolemia (online-only Data Supplement), several studies report reduced CBF velocity (a surrogate measure of blood flow) in the middle cerebral artery, assessed by transcranial Doppler ultrasonography (TCD) in healthy volunteers.^10–13^ Studies examining the dynamic regulation of CBF in hypertension in response to changes in BP show mixed results, with some showing preserved ability to regulate flow compared with normotensive individuals^14,15^ and others showing an impairment of regulation.^16,17^ Given that hypertension is associated with structural changes in response to increased transmural pressure, such as arterial stiffening and atherosclerosis,^18–20^ it remains unclear whether the arterial remodeling may contribute to the altered CBF regulation. Any impairment in the regulation of CBF and lower perfusion at rest may risk oxygen deprivation and ischemia in hypertensive patients.

In healthy volunteers, regulation of CBF is crucially dependent on alterations in cardiac output (CO).^21,22^ However, whether CO plays a role in maintaining CBF in patients with hypertension is unknown. Thus, the main aim of this study was to assess whether the CBF response to reductions in CO is impaired in people with hypertension compared with an age-matched normotensive cohort. We recruited 2 groups of hypertensive patients, those with either treated controlled (controlled hypertensives) or uncontrolled BP (uncontrolled hypertensives), to assess whether these groups respond differently to reductions in CO. We hypothesized that patients with hypertension would have a larger reduction in CBF in response to a decrease in CO induced by LBNP. We also hypothesized that because uHTNs are likely to have more adverse arterial remodeling versus cHTN,^8,19,20,23^ this group would have a larger decline in CBF during a reduction in CO.

## Methods

This study was approved by the UK National Research Ethics Committee (reference: 15/SW/0176) and the Institutional Research and Innovation Department at the University Hospitals Bristol NHS Foundation Trust. All methods conformed to the Declaration of Helsinki, as well as local and UK national guidelines. Data and analysis is available on request from https://data.bris.ac.uk/data/.

### Participants and Screening

A total of 39 eligible men and women were recruited for the study, of which 13 were normotensive, 13 were hypertensive with BP controlled by medication (cHTN), and 13 were uncontrolled hypertensives (uHTN; Table). Participants were age matched and grouped based on daytime ambulatory BP averaged over at least 14 measurements, in accordance with 2018 European Guidelines for diagnosis of hypertension. The normotensive and cHTN groups were defined as a daytime ambulatory BP of <135/85 mmHg. uHTN was defined as a daytime ambulatory BP of >135/85 mmHg, either without or despite being prescribed antihypertensive medication. The uHTN group was included to investigate the effect of raised resting BP.

**Table.**
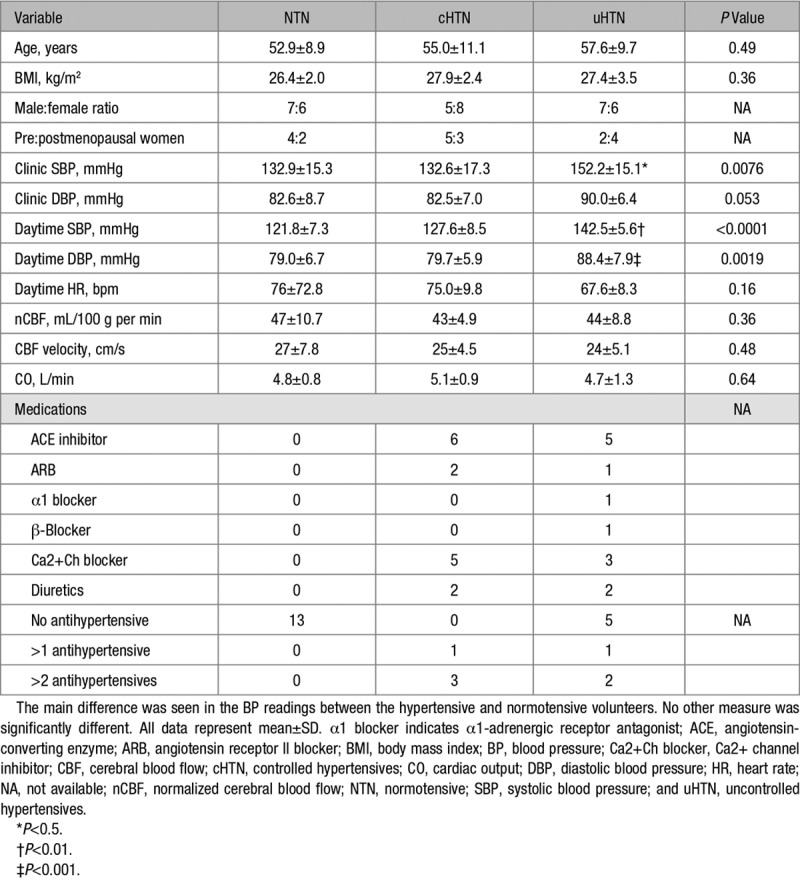
Demographics

All participants gave written informed consent. Participant screening procedures and eligibility is detailed in the online-only Data Supplement.

### Lower Body Negative Pressure

A magnetic resonance (MR) imaging compatible polycarbonate box was fitted to encompass the lower torso and legs (online-only Data Supplement). A broad polyurethane skirt was wrapped around the waist and secured over the box providing an airtight seal. The participants were positioned with their feet flat against the end of the box to prevent the participant being pulled further into the box by the suction. A commercially available vacuum cleaner with continuous variable power (Sebo Airbelt K1 1800 W) was attached to the box. The negative pressure achieved inside the box was monitored with an MR safe pressure gauge (SM Gauge) continuously indicating the pressure within the box (relative to atmospheric).

The LBNP protocol was graded at −20, −40, and −50 mmHg in descending order. Participants were maintained at each level of LBNP for 1 minute before imaging. Each phase-contrast scan took ≈5.5 minutes.

### Physiological Monitoring

An automated sphygmomanometer measured BP and heart rate (HR) every 1.5 to 2 minutes during each phase-contrast acquisition. An average of the measurements was taken for each level of LBNP. A nasal cannula was connected to a calibrated capnograph (Capstar100; CWE, Inc) to measure the percentage of expired end-tidal CO_2_. Breathing rate was not monitored in the study. However, a suspected change to mouth breathing was considered when little-to-no end-tidal CO_2_ was detected; this was seen as a near-flat line of the end-tidal CO_2_ trace.

### MR Imaging

All MR images were acquired during free breathing at 1.5T (Siemens Avanto Magnetom, Siemens Healthineers).

#### Phase-Contrast MR Angiography

ECG-gated phase-contrast MR angiography was used to measure (1) CBF in the basilar and internal carotid arteries, in the transverse plane perpendicular to the internal carotid arteries at the level of the basilar artery, followed by (2) flow in the ascending aorta in the transverse plane, at the level of the main pulmonary trunk (i.e., CO). All flow imaging was performed at isocenter. Examples of flow data in the intracranial arteries and the aorta are shown in Figure S1 in the online-only Data Supplement.

#### Time-of-Flight Angiogram

The time-of-flight angiogram was covering the superior vertebral arteries and the entirety of the Circle of Willis. Time-of-flight images were used primarily to position the phase-contrast images for the intracerebral flow measurements. However, a 3-slice localizer with slice thickness of 5 mm was used for the repositioning of the flow slices during LBNP to ensure a consistent positioning regardless of movement of the subject due to suction. An example angiogram is shown in Figure S1.

#### T1-MPRAGE

A 3-dimensional T1-weighted scan (T1-MPRAGE) was acquired in the transverse plane.

### Statistical Analysis

MR image analysis is outlined in the online-only Data Supplement.

We calculated that a sample size of 11 in each group provides a power of 80% to find a 17% difference (estimated based on^24^ Abstract) in resting CBF between the uHTN and normotensive groups with a 2-sided α of 0.05.

Inter- and intraobserver variability data are available in the online-only Data Supplement.

Mean arterial BP (MAP) was calculated as MAP=diastolic BP (DBP)+ 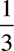 (pulse pressure). Total peripheral resistance (TPR) was estimated as 
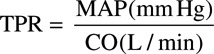


Cerebrovascular conductance was calculated as:


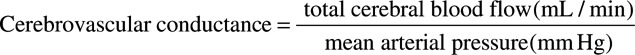


Analyses were completed in Prism v7 (GraphPad Software) unless otherwise stated.

Normality of distribution in the data was assessed by D’Agostino-Pearson. CO did not pass the test of normality and was transformed by a log_10_ transformation.

Mean differences between the groups during LBNP were analyzed using mixed-model ANOVA and compared using Tukey multiple comparisons with respect to resting values. Mean differences between the groups at rest were analyzed by a 1-way ANOVA and Bonferroni correction for multiple comparison. Analysis by sex is presented in the online-only Data Supplement.

A 3-way ANOVA with factors artery, LBNP, and group was performed to assess potential interactions between the arteries and groups during LBNP (SPSS v23; IBM).

Vascular remodeling and structural incidents related to vascular disease are included in the online-only Data Supplement. The data are reported as mean and SD. α was set at 0.05.

## Results

All participants completed the experimental protocol. One participant who, due to presyncopal symptoms, was unable to tolerate LBNP of −50 mmHg, had incomplete data for aortic flow and was excluded from all assessments of CO. In 9 participants, end-tidal CO_2_ was not reliably recorded because of technical difficulties.^9^ These participants were removed from the CO_2_ analysis. Incidence of stenosis can be found in Table S1 presented in the online-only Data Supplement. Only mild stenosis was observed. Interestingly, 4 of the normotensives showed stenosis versus 3 in uHTN and 0 in cHTN.

### Participants

The participant demographics are summarized in the Table. There were no differences in the average age, body mass index, or sex ratio between groups. Clinic and ambulatory BPs were higher in the uHTN compared with normotensive and cHTN (1-way ANOVA; clinic systolic blood pressure [SBP], *P*=0.0076; ambulatory SBP, *P*<0.0001; ambulatory DBP, *P*=0.0019) but not clinic DBP (*P*=0.05). There was no difference in BP between normotensive and cHTN (clinic SBP, *P*>0.99; clinic DBP, *P*>0.99; ambulatory SBP, *P*=0.15; ambulatory DBP, *P*>0.99).

### Physiological Responses

**Figure 1.**
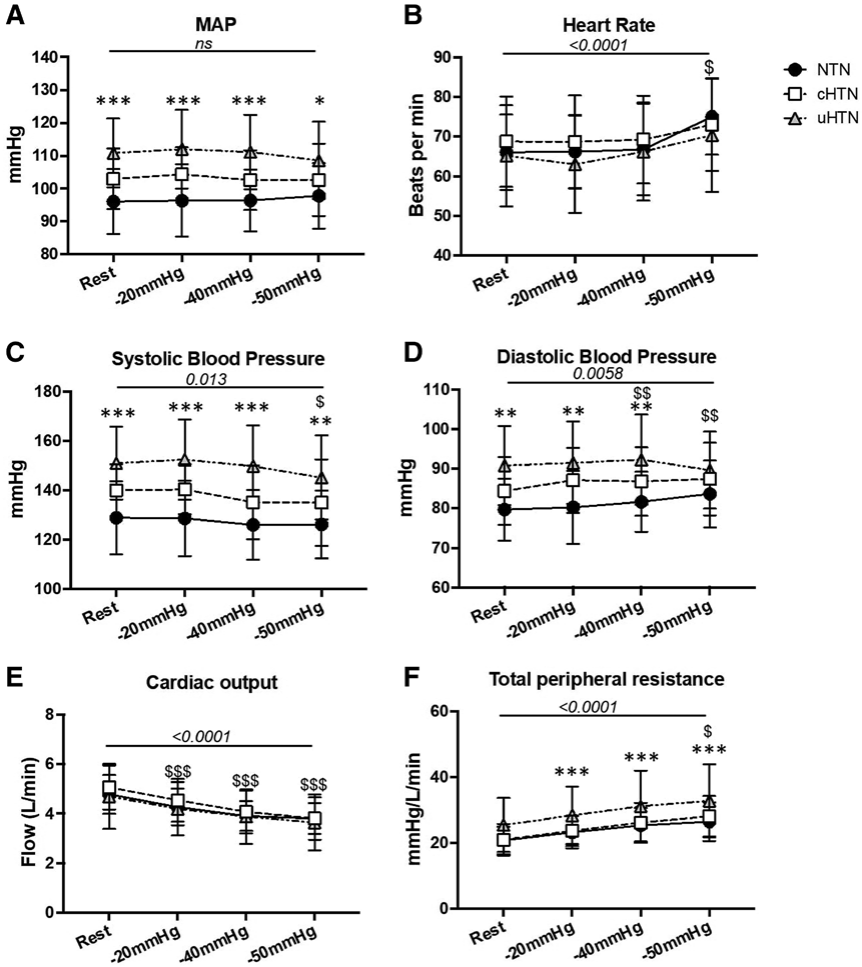
Physiological responses to lower body negative pressure (LBNP). **A**, Mean arterial blood pressure (MAP; mmHg) was unchanged during LBNP but higher in the uncontrolled hypertensives (uHTN). **B**, Heart rate (beats per minute) increased during LBNP at −50 mmHg with no difference between the groups. **C**, Systolic blood pressure (mmHg) showed a significant reduction at −50 mmHg, as well as a consistently higher pressure in the uHTN compared with normotensives (NTN). **D**, Diastolic blood pressure (mmHg) was significantly increased at −40 and −50 mmHg LBNP, as well as being higher in the uHTN group. **E**, cardiac output (L/min) decreased during LBNP with no difference between the groups. **F**, Total peripheral resistance (mmHg/L per min) increased with LBNP in all groups and was persistently higher in the uHTN, *P* above the line refers to ANOVA main effect of LBNP. * & $ *P*<0.05, ** & $$ *P*<0.01, *** & $$$ *P*<0.001 are multiple comparison *P* where * is NTN vs uHTN and $ LBNP vs rest. ns indicates nonsignificant.

Figure 1 shows BP, HR, TPR, and CO responses to LBNP. There was an increase in HR at a LBNP of −50 mmHg only (5.4% of the total variance, *P*<0.0001) with no difference between the groups (1.7% of the total variation, *P*=0.7). There was no change in MAP with LBNP (0.16% of the total variation, *P*=0.3), nor an interaction of the change in MAP between groups and LBNP (0.49% of the total variation, *P*=0.09). Overall, SBP was significantly lowered by LBNP (1.48% of the total variation, *P*=0.0013), at −50 mmHg LBNP only when compared with rest (*P*=0.013). There was no interaction effect between LBNP and group on SBP (0.3% of the total variation, *P*=0.7). DBP increased with LBNP (0.7% of the total variation, *P*=0.0058), at −40 mmHg (*P*=0.01) and −50 mmHg (*P*=0.01). DBP was significantly different between groups (16.75% of the total variation, *P*=0.03), and there was an interaction of DBP between LBNP and group (0.98% of the total variation, *P*=0.0054). DBP significantly increased in the normotensive group at −50 mmHg (*P*=0.0024) and in the cHTN group (*P*=0.034) but not the uHTN (*P*=0.9). Between-group Dunnett post hoc comparisons showed a significant difference for DBP between the normotensive and uHTN only (*P*=0.01).

LBNP increased TPR (11.25% of the total variation, *P*<0.0001). There was no interaction between the change in TPR during LBNP and group (0.14% of the total variation, *P*=0.90). There was a reduction in end-tidal CO_2_ with LBNP (5.4% of the variation, *P*<0.0001; n=30). Compared with rest, the reduction was 0.08% at −20 mmHg (*P*=0.3), 0.2% at −40 mmHg (*P*=0.0009) and 0.3% at -50 mmHg (*P*<0.0001). Regarding end-tidal CO_2_, there was no difference between groups (1.0% of the variation, *P*=0.8) or interaction between LBNP and group (0.16% of the variation, *P*=0.9). However, a change from nasal to oral breathing was observed at −50 mmHg LBNP. Unfortunately, we did not measure breathing rate, and as such, no data are available on the minute ventilation.

### Cardiac Output

There was a reduction in CO during LBNP (17.99% of the variation, *P*<0.0001), with no difference between the groups (1.28% of the total variation, *P*=0.74) or interaction between LBNP and group (0.28% of the total variation, *P*=0.53). CO was reduced compared with rest at −20 (*P*<0.0001), −40 (*P*<0.0001), and −50 mmHg (*P*<0.0001) in all participants (Figure 1).

### Cerebral Blood Flow

**Figure 2.**
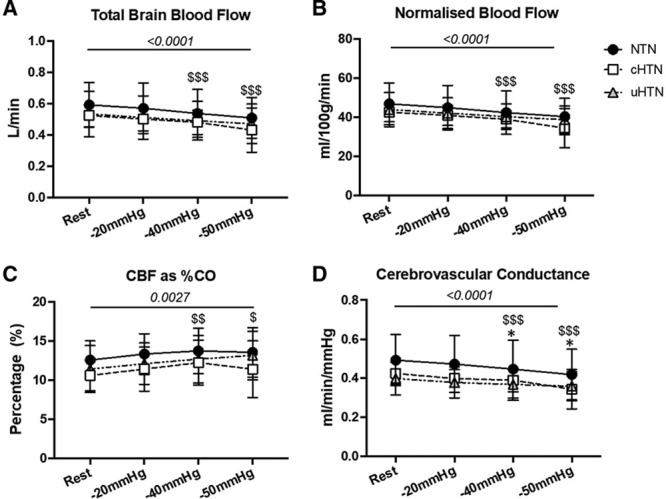
Cerebral blood flow (CBF) during lower body negative pressure (LBNP) to −50 mmHg. **A**, total cerebral blood flow (tCBF) (L/min) decreased with LBNP. **B**, Normalized CBF (nCBF) decreased with LBNP. **C**, Percentage of the cardiac output (CO) to the brain (%) increased with LBNP. **D**, Cerebrovascular conductance (mL/min per mmHg) decreased with LBNP and showed a lower conductance in the uncontrolled hypertensives (uHTN) at −40 and −50 mmHg compared with the normotensives (NTN). *P* above the line refers to ANOVA main effect of LBNP. * & $ *P*<0.05, ** & $$ *P*<0.01, *** & $$$ *P*<0.001 are multiple comparison *P* where * is NTN vs uHTN and $ LBNP vs rest.

Figure 2 shows total CBF (tCBF), normalized CBF, and cerebrovascular conductance during LBNP. Resting normalized CBF was similar between the groups (Table). Normalized CBF (8.2% of the variation, *P*<0.0001) and blood flow velocity (2% of the total variation, *P*<0.0001) were reduced by LBNP. Post hoc tests revealed a significant reduction in normalized CBF at −40 (*P*<0.0001) and −50 mmHg (*P*<0.0001) but not at −20 mmHg (*P*=0.13). There was no interaction between LBNP and group (0.4% of the variation, *P*=0.78).

LBNP reduced CBF in all 3 intracranial arteries measured (basilar and internal carotid arteries, *P*<0.0001) with no interaction between LBNP and artery (*P*=0.64) or between LBNP, artery, and group (*P*=0.92; Figure S3).

There was a reduction in cerebrovascular conductance during LBNP (4.9% of the total variation, *P*<0.0001), with no difference between the groups (11.35% of the total variation, *P*=0.07) or interaction between LBNP and group (0.5% of the total variation, *P*=0.47). Cerebrovascular conductance was reduced at −40 (*P*=0.0004) and −50 mmHg (*P*<0.0001) but not at −20 mmHg (*P*=0.053). No correlation was observed between the change in end-tidal CO_2_ and the change in tCBF (r=0.07, *P*=0.7116).

On average, tCBF reduced by −3.9% at −20 mmHg, −8.2% at −40 mmHg, and by −14.6% at −50 mmHg LBNP from resting values. There was an increase (3.2% of the total variation, *P*=0.0027; n=38) in the percentage of the CO that was distributed to the brain (i.e., percentage of CO measured as tCBF at any given level of LBNP). This difference amounted to 0.75% at −20 mmHg (*P*=0.12), 1.35% at −40 mmHg (*P*=0.0015), and 1.15% at −50 mmHg (*P*=0.0081). There was no difference between the groups (7.1% of the total variation, *P*=0.17) and no interaction between the change in the percentage of the CO going to the brain during LBNP and group (0.5% of the total variation, *P*=0.86).

## Discussion

The novel finding in this study was that despite an absence of a change in MAP, tCBF decreased with LBNP-mediated reductions in CO by a comparable value in normotensive, uHTN, and cHTN. Thus, both normotensive and hypertensive volunteers appear equally unable to maintain tCBF during simulated hypovolemia.

In the present study, the baroreflex-mediated increase in HR during LBNP was not sufficient to compensate for the reduction in CO. MAP was unchanged in all groups during LBNP, explained by an increase in TPR. Thus, cerebral perfusion pressure appears to be maintained during simulated hypovolemia despite the reduction in CO. These data are consistent with previous studies in normotensive subjects where middle cerebral artery CBF velocity was reduced during LBNP with unchanged MAP.^25,26^

### Methodological Considerations

Previous studies have largely relied on TCD to estimate changes in blood flow in cerebral vessels. TCD measures the mean velocity as a surrogate of flow and assumes a static caliber of the vessel. It is dependent on the insonation angle, which must be perpendicular to the center of the vessel, and on the cross-sectional area of the vessel, which is assumed to be constant. Although it has been argued that the change in the cross-sectional area of the artery during BP challenges and the pulsation during the cardiac cycle are negligible,^14,27^ this assumption may not hold true when comparing arteries in hypertensive individuals to those of normotensives. By comparison, MR phase-contrast angiography offers a repeatable and more reliable means for measuring blood flow.^28,29^ Leung et al^28^ proposed that phase-contrast angiography is more appropriate for studies wishing to quantify other derived measures such as cerebrovascular resistance, as we have done herein.

Additionally, MR phase-contrast imaging allows simultaneous measurement of blood flow in the intracranial parts of the internal carotid and basilar arteries, which are not easily accessible with TCD, and provides a measure of global CBF. However, TCD provides information about dynamic changes in blood flow velocity that cannot be provided by magnetic resonance imaging. While both TCD and phase-contrast angiography depend on the angle with respect to the vessel at which the measurement is taken, phase-contrast angiography offers a more reliable and verifiable positioning of the measurement compared with TCD, for example, by the acquisition of linked time-of-flight angiograms, as was done in this study.

The present study was not designed to look at the effect of antihypertensive medication on CBF regulation. A rapid adaptation of CBF in response to antihypertensive therapy has been reported.^23^ This represents a significant limitation to the present study. It is important to note that both the cHTN and uHTN groups presented with a variety of antihypertensive medications, dosages, and combinations of ≥2 treatments used in conjunction. Nonetheless, the present study assessed these groups to explore the effect of controlled and uncontrolled BP, rather than the effect of treatment on CBF. Therefore, these groups largely reflect the spectrum of patients typically found in clinics.

### Cardiovascular Responses to LBNP

The decrease in CO was similar to that observed after 25 minutes of prolonged standing in the same position, where CO was reduced by 1.4 L/min.^30^ This is similar to the 1.1 L/min reduction in CO observed at −50 mmHg LBNP; indicating that the change in CO in our study represents a physiologically relevant stressor to the cardiovascular system. During LBNP, cerebrovascular conductance decreased, suggesting that vasoconstriction occurred in the intracranial cerebral circulation. This is in contrast to the increase in cerebrovascular conductance reported previously in healthy volunteers.^31^ Consistent with the present results, several other studies have shown a decrease in cerebrovascular conductance during LBNP in healthy volunteers.^11,12,21,25,32^ One limitation of cerebrovascular conductance estimation is that conductance is inferred from peripheral arterial pressure measurements. Measuring intraarterial pressure in the cerebral circulation is technically challenging and currently not possible to achieve noninvasively. No studies have measured the linearity or relationship between brachial pressure and intracerebral BP in vivo. However, in the supine position, hydrostatic column effects are minimal, and, therefore, the head perfusion pressure is likely to be similar to brachial pressure.^33^

We demonstrated a reduction end-tidal CO_2_ during LBNP that may have contributed to the reduced cerebral vascular conductance. Although a reduction in end-tidal CO_2_ during LBNP has been reported before,^10,11^ it is important to note that we measured end-tidal CO_2_ through a nasal cannula and did not monitor respiratory rate. If the participants were breathing through the mouth rather than nose during increased LBNP, this would explain the decrease in the measured end-tidal CO_2_. Alternatively, the reduction in end-tidal CO_2_ may be explained by the fall in CO. Regardless, even with CO_2_ clamped at resting levels, a reduction in CBF velocity has been measured with LBNP.^34^

### Brain Blood Flow During LBNP

Previous reports have pointed to a lower resting CBF in patients with hypertension.^2,4^ We did not replicate this finding; however, a difference in sample size or methodological differences could explain this discrepancy. Warnert et al^4^ used phase-contrast angiography with a greater spatial resolution (voxel size, 0.9×0.9×2 mm^3^) in >100 participants but acquired only 9 reconstructed phases over the cardiac cycle. Therefore, while Warnert et al^4^ had good spatial resolution and thus reliable measurements of vessel diameter, 9 frames per cardiac cycle equates to an effective temporal resolution of ≈111 ms (assuming a HR of 60 beats per minute), which will inescapably lead to undersampling.^35^ By contrast, the present study acquired phase-contrast flow with 100 reconstructed phases over the cardiac cycle, equating to an effective temporal resolution of ≈10 ms. The 10-fold higher temporal resolution of the current study allows for a more detailed and reliable characterization of flow throughout the cardiac cycle. Lastly, the current study sample comprised middle-aged subjects who may show less pronounced differences in resting CBF, when compared with an older demographic with more advanced vascular disease.^3^

Three main theories on how the body maintains optimal brain blood flow are relevant and should be considered. First, proportional blood distribution states that blood flow to each organ is a fixed percentage of the CO, and each organ’s share is based on their metabolic demand.^22,36^ The present study does not support this theory given that the percentage of the CO going to the brain increased (albeit by 1%) with LBNP. Second, based on the principles of Poiseuille law, the perfusion of any organ depends on the arterial pressure and vascular resistance. In the present study, the lower flow and conductance observed in response to a reduced CO may have been compensated for by vasoconstriction of the cerebral circulation (e.g., triggered by hypocapnia) thereby maintaining perfusion pressure. Third, as the regulation of CBF is critical for maintaining constant nutrient and oxygen supply to the brain, a brain-specific theory for the regulation of blood flow, referred to as the selfish brain hypothesis, has been proposed. According to this theory, the body prioritizes CBF and adjusts peripheral BP to adjust flow to the brain to maintain constant perfusion.^37^ The theory further proposes that hypertension is caused by a reduction in brain stem perfusion driving up BP to normalize the perfusion. Eventually, the BP becomes insufficient to compensate for the lowered perfusion, leading to a permanently lowered CBF.^4^

The current study suggests that irrespective of high BP, the brain is able to tolerate a reduction in tCBF when CO falls at rest. The fact that CBF fell by −14.6% at −50 mmHg LBNP supports this idea. However, this claim may be challenged by the increase seen in the percentage of the CO going to the brain, suggestive of a degree of priority given to brain perfusion. Indeed, we saw an increase of ≈1% at −50 mmHg LBNP. This supports the selfish brain hypothesis in that CBF is given priority through an increase in the proportional distribution ably assisted by increasing peripheral vascular resistance to maintain arterial pressure. However, at −40 mmHg where CBF was reduced by an average of −8.2%, an equivalent increase in the percentage of the CO going to the brain was observed (i.e., a mere 1%), suggesting a reserve would still be available at this level given the steady decline in CO, unchanged MAP, and HR. Furthermore, we did not measure blood flow to any other organ and, therefore, do not have information on whether the redistribution of blood flow may be unique to the brain.

### Perspectives

Reductions in CBF caused by conditions that lower CO in populations with already low resting CBF and extensive microvascular disease may put the cerebral circulation at risk of hypoperfusion and ischemic events.

Given that MAP was unchanged during LBNP, this may suggest that, so long as perfusion pressure can be maintained, a reduction in CBF as a consequence of reduced preload is tolerated. As the brain has a limited capacity for metabolic substrate storage,^38^ the lower CBF must still be able to meet the metabolic demands. It is, therefore, of interest to know whether a cognitive task would increase CBF under conditions of LBNP or whether the performance on such a task would suffer. Under resting conditions, it may be assumed that sufficient CBF was available to meet demand. This can be achieved either through a change in the extraction fraction of metabolic substrate from the blood during perfusion, for example, by the slowing of blood flow as seen in the present study, or by an abundance of blood flow at rest. Importantly, these mechanisms are likely to be dependent on the perfusion pressure to maintain the substrate exchange across the blood-brain barrier. Future studies should focus on measuring the perfusion of the brain with respect to oxygen extraction during a hypovolemic challenge to better understand whether lowering of CBF has functional consequences.

### Conclusions

In summary, the study shows that in middle-aged men and women, tCBF decreases when CO decreases. Since MAP was maintained during LBNP, this suggests that cerebral perfusion pressure was unchanged despite a reduction in tCBF. There was no difference in the decline in CBF between normotensive and hypertension groups, indicating that hypertension does not impact the regulation of CBF during simulated hypovolemia.

## Acknowledgments

We would like to acknowledge the support received from the Bristol Heart Institute radiographers to conduct this study. Further, we wish to acknowledge the research nurses who have helped with the recruitment and eligibility screening of the participants. Finally, we wish to thank the participants who volunteered their time to take part in this study.

## Sources of Funding

This study was supported by the Wellcome Trust and the Bristol National Institute for Health Research (NIHR) Biomedical Research Centre. The views expressed are those of the authors and not necessarily those of the National Health Service, NIHR, or the Department of Health and Social Care.

## Disclosures

None.

## Supplementary Material

## Novelty and Significance

### What Is New?

- We demonstrate that blood flow to the brain reduces when the amount of blood pumped by the heart is reduced by simulated blood loss. And this reduction in blood flow to the brain is similar in people with high blood pressure to those with normal blood pressure.

### What Is Relevant?

- Understanding the effect of blood pressure on brain blood flow is important because poor regulation of brain blood flow is linked to an increased risk of stroke and dementia. This study suggests that cardiac output can change brain blood flow even without a change in blood pressure.

### Summary

Simulated blood loss lowers cardiac output without lowering blood pressure and results in lower blood flow to the brain. Blood flow and blood flow regulation are similar in people with high and those with normal blood pressures.
