# Supplementary material for: Cerebral Blood Flow Response to Simulated Hypovolemia in Essential Hypertension: A Magnetic Resonance Imaging Study
Source: Hypertension. 2019 Oct 28;74(6):1391–8. doi: 10.1161/HYPERTENSIONAHA.119.13229 (PMC7069391; doi:10.1161/HYPERTENSIONAHA.119.13229)
Supplement: Supplementary file 1 [file hyp-74-1391-s001.pdf]

Online supplement

Supplemental material for:

Cerebral blood flow response to simulated hypovolaemia in essential hypertension: an MRI study

Neumann S, BSc(Hons), MSc, PhD[1,3], Burchell AE, MA, MBBCh, MRCP, PhD[2], Rodrigues JCL, BSc(Hons), MBChB(Hons), MRCP, FRCR, PhD[2,4], Lawton CB, BSc(Hns) PGc[2], Burden D BSc(Hons) [2], Underhill M BSc(Hons)[2], Kobetić MD BSc(Hons), MSc, MBChB[3], Adams ZH, BSc(Hons)[1], Brooks JCW, BSc, PhD[1], Nightingale AK, MA, MBBChir, FRCP, MD[1,2], Paton JFR BSc, PhD[1,5], Hamilton MCK, MBChB, MRCP FRCR*[2], Hart EC, BSc, PhD*[1]

Screening and eligibility

Participants were screened by 12 lead ECG, a Siemens Multistix urine dipstick, clinic and ambulatory blood pressure monitoring, as well as height, weight, hip circumference and testing for orthostatic intolerance.

Participants were excluded if they were diagnosed with any cardiovascular (other than hypertension and hypertensive left ventricular hypertrophy), autonomic, respiratory, renal, cognitive or immunological disorders, BMI >35, or if they have a hip circumference >120cm due to the practical constraint of the lower body negative pressure chamber.

MR imaging parameters

Cerebral images were acquired with a 12 channel Head Matrix coil (Model 07577732, Siemens Heathineers), whilst cardiac images were acquired with an 8 channel Body Matrix coil (Model 07579555, Siemens Heathineers).

Phase contrast

The acquisition details of the phase contrast angiography were as follows: echo time (TE)/effective temporal resolution 2.61/10.95ms, flip angle 30 degrees, field of view (FoV) 250mm, phase FoV 75%, matrix: 256 x 100pixels, slice thickness 5mm, phase encoding direction R/L, voxel dimensions 1.0 x 1.0 x 5.0mm, number of phase reconstructions 100, maximum velocity encoding, (VENC) 150 cm/s, 3 averages.

Time of flight angiogram

The acquisition details of the Time of Flight angiogram was as follows: TE/repetition time (TR): 7/23ms, flip angle 25 degrees, phase encoding R/L, FoV: 200mm, phase FoV 94%, matrix: 256 x 94 pixels, slice resolution 85%, slice thickness: 0.8mm, voxel dimensions 0.8 x0.8 x0.8 mm isotropic.

T1-MPRAGE

The T1-MPRAGE was acquired with TE/TI/TR = 3.37/1100/1900ms, flip angle 15 degrees, FoV 256mm, phase FoV 75%, matrix 256 x 100pixels, GRAPPA acceleration factor 2, slices per slab 176 with slice resolution 80%, voxel size 1.0x1.0x1.0mm isotropic, phase encoding R/L.

MR image analysis

The arteries were contoured in each frame of reconstructed flow from the smoothed magnitude images (see supplemental Figure S1). Mean flow velocity and total flow were quantified in each vessel using semi-automated Siemens software (Argus, Siemens Healthineers). Total CBF (tCBF) was calculated by summing the flow in the basilar and the internal carotid arteries. CBF was normalised to total brain tissue volume (nCBF) and presented as millilitres per 100g of tissue per minute. The brain tissue volumes were calculated from the T1-MPRAGE using FAST segmentation (FSL software; [1]) and the number of voxels for each tissue type quantified using FSLSTATS. The volumes were converted to grams assuming that 1cm^3 brain tissue weighs 1.036 grams [2].

About Lower Body Negative Pressure

Lower body negative pressure (LBNP) is a technique used to manipulate central blood volume, and has been validated as a model for haemorrhage in the baboon [3]. Typically, the lower body is encased in an airtight chamber from the level of the waist or iliac crest and a suction device used to manipulate the pressure within the chamber relative to athmospheric pressure.

LBNP is known to cause a large variety of physiological changes, including decreased central venous pressure, reduced preload of the heart, reduced stroke volume, reduced cardiac output and baro-reflex induced increases in heart rate, total peripheral resistance, muscle sympathetic nerve activity and the release of vasoactive and volume regulating endocrine factors, which are typically mimicking the effects of hypovolemia due to blood loss [4]. For example, Rickards *et al.* [5] showed that LBNP at -45mmHg and blood loss of up to 1000mL resulted in similar reductions in cerebral blood flow velocity and neither induced changes in MAP. Hanson *et al.* [6] showed that the reduction in stroke distance at -20mmHg LBNP was equivalent to a blood loss of ~450mL. Finally, Hinjosa-Laborde *et al.* [3] suggested that LBNP at approximately -20mmHg equated to a blood loss of ~6.25% of the total blood volume, -40mmHg LBNP equated to a blood loss of ~12.5% of the total blood volume, and -50mmHg LBNP equated to a blood loss of ~18.75% of the total blood volume in the baboon model. Furthermore, Hinjosa-Laborde *et al.* [3]showed similar physiological reduction in pulse pressure, central venous pressure, systolic blood pressure, heart rate, stroke volume, cardiac output, vascular resistance, as well as similar changes in renin activity, blood bicarbonate, blood lactate, blood pH, partial pressure of oxygen, partial pressure of CO_2, blood osmolarity, blood sodium levels, blood urea nitrogen, and glucose content between LBNP and blood loss.

Intra- and Inter-observer variability

Inter-observer variability of flow measurements from the ICA was performed by an independent and blinded researcher (SN and MDK) on 10 randomly selected participants. Intra-observer variability of the same ICA measurements was performed in 20 randomly selected participants.

The interclass correlation for the CBF inter-observer variability was 0.963 (95% CI: 0.862-0.991, $p<0.0001$) for single measures and 0.981 (95% CI: 0.926-0.995, $p<0.0001$) for average measures. For the average velocity the inter-observer variability interclass correlation coefficient was 0.996 (95% CI: 0.847-0.999, $p<0.0001$) for single measures and 0.998 (95% CI: 0.917-1.00, $p<0.0001$) for average measures.

The interclass correlation coefficient for the CBF intra-observer variability was 0.984 (95% CI: 0.930-0.995, $p<0.0001$) for single measures and 0.992 (95% CI: 0.964-0.997, $p<0.0001$) for average measures. For the average velocity data in the ICA the inter-observer variability interclass correlation coefficient was 0.996 (95% CI: 0.985-0.999, $p<0.0001$) for single measures and 0.998 (95% CI: 0.992-0.999, $p<0.0001$) for average measures.

Arterial Stenosis

As a surrogate marker of vascular remodelling, arterial stenosis was assessed from the time of flight angiograms.

Methods

A trained radiologist (>8 years experience) assessed the time of flight angiograms evaluating the presence of internal carotid and vertebral artery stenosis. The data was reviewed in 3 orthogonal multiplanar-reformatted planes with cross-referencing of images. Maximum intensity projection images were generated and reviewed. Vessels were interrogated for the presence of focal atherosclerotic stenosis. Where present, a measurement of percentage area stenosis relative to adjacent normal calibre proximal and distal vessel was made.

Results

Stenosis observed in the sample is shown in Table S1 with the rates of stenosis in each group.

Data by Sex

Given that there was no difference between the groups, all participants were pooled and grouped according to sex (male versus female). There was no difference between the sxes for tCBF during LBNP (p=0.28), MAP during LBNP (p=0.19), nor in the percentage of the CO diverted to the tCBF during LBNP (p=0.22). However, CO was affected differently in male and female volunteers (p=0.01, 11% of the total variation) and showed an interaction between LBNP and sex (p=0.01, 0.5% of the total variation). *Post hoc* CO was significantly lower in female volunteers at -40mmHg (p=0.045) and -50mmHg (p=0.0055) LBNP. This data is shown in S5 below.

Supplemental references

1. Zhang Y, Brady M, Smith S. Segmentation of brain MR images through a hidden Markov random field model and the expectation-maximization algorithm. *IEEE Trans Med Imag* 2001;20:45-57

2. Hofmann MA. The fractal geometry of convoluted brains. *J Hirnforschung* 1991;32:103-111

3. Hinjosa-Laborde C, Shade RE, Muniz GW, Bauer C, Goei KA, Pidcoke HF, Chung KK, Cap AP, Convertino VA. Validation of lower body negative pressure as an experimental model of haemorrhage *J Appl Physiol (1985):* 2014;116(4):406-415

4. Cooke WH, Ryan KL, Convertino VA. Lower body negative pressure as a model to study progression to acute hemorrhagic shock in humans. J Appl Physiol 2004;96:1249–1261

5. Rickards CA, Johnson BD, Harvey RE, Convertino VA, Joyner MJ, Barnes JN. Cerebral blood flow velocity regulation during progressive blood loss compared with lower body negative pressure in humans. *J Appl Physiol* 1995;119(6):677-685

6. Hanson JM, Van Hoeyweghen R, Kirkman E, Thomas A, Horan MA. Use of stroke distance in the early detection of simulated blood loss *J Trauma* 1998;44(1):128-134

Table S1: Arterial stenosis

Location of Stenosis	NTN	uHTN	cHTN
Left focal stenosis (mild 1-25%)	1	1	0
Right focal stenosis (mild 1-25%)	2	2	0
Left vertebral focal stenosis (mild 1-25%)	1	0	0
Right vertebral focal stenosis (mild 1-25%)	0	0	0

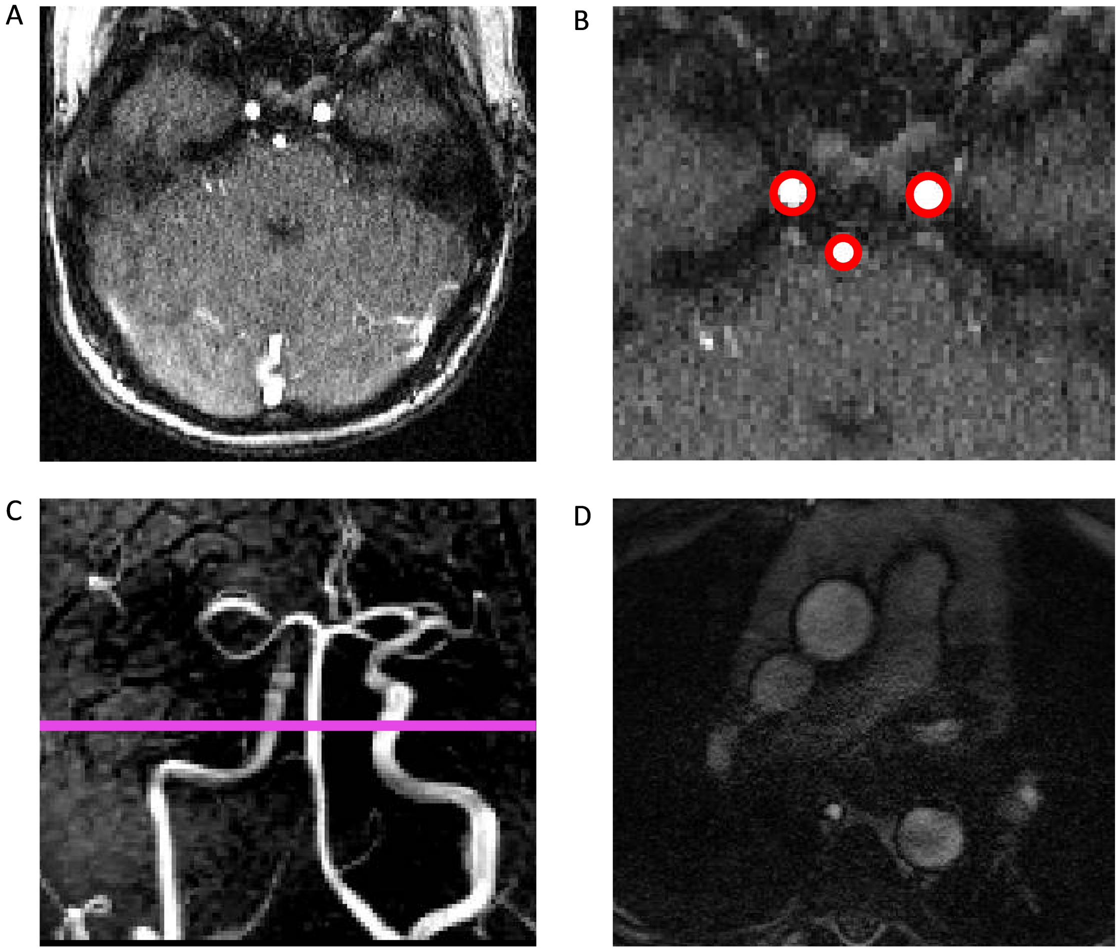

Figure S1: Examples of MR data collected, A) Phase contrast image of the internal carotid and basilar arteries **B)** Zoomed view of the phase contrast image of the internal carotid and basilar arteries seen in A illustrating the contour lines for each vessel in red **C)** Time of flight angiogram showing the imaging plane (magenta) for the phase contrast images in A (& B). **D)** Phase contrast image in the ascending aorta at the levels of the pulmonary trunk.

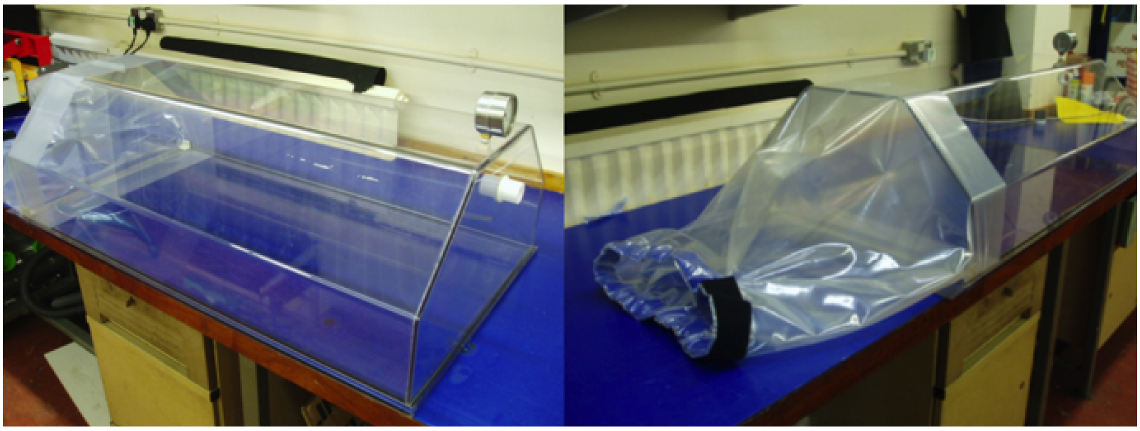

Figure S2: Picture of the lower body negative pressure chamber

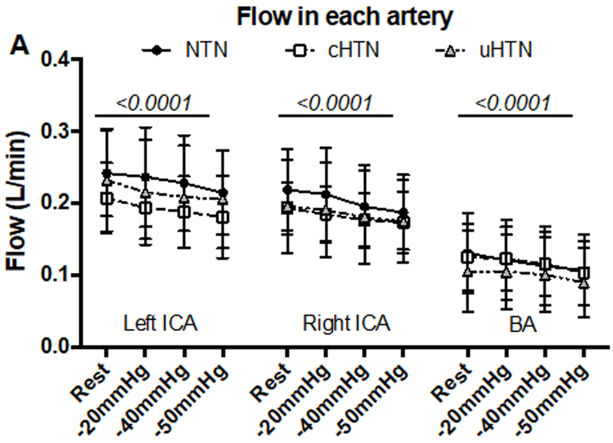

B

		-20mmHg	-40mmHg	-50mmHg
Left ICA	NTN	ns	$	$$$
	cHTN	ns	$	$$
	uHTN	$	$$	$$$
Right ICA	NTN	ns	$$$	$$$
	cHTN	ns	ns	$
	uHTN	ns	$	$$
BA	NTN	ns	$$	$$$
	cHTN	ns	ns	$$
	uHTN	ns	ns	$

Figure S3: Flow in each intracranial artery, A) Blood flow in each intracranial artery (L/min), **B)** Table showing the post hoc tests measuring difference in flow during LBNP in each artery for each group.

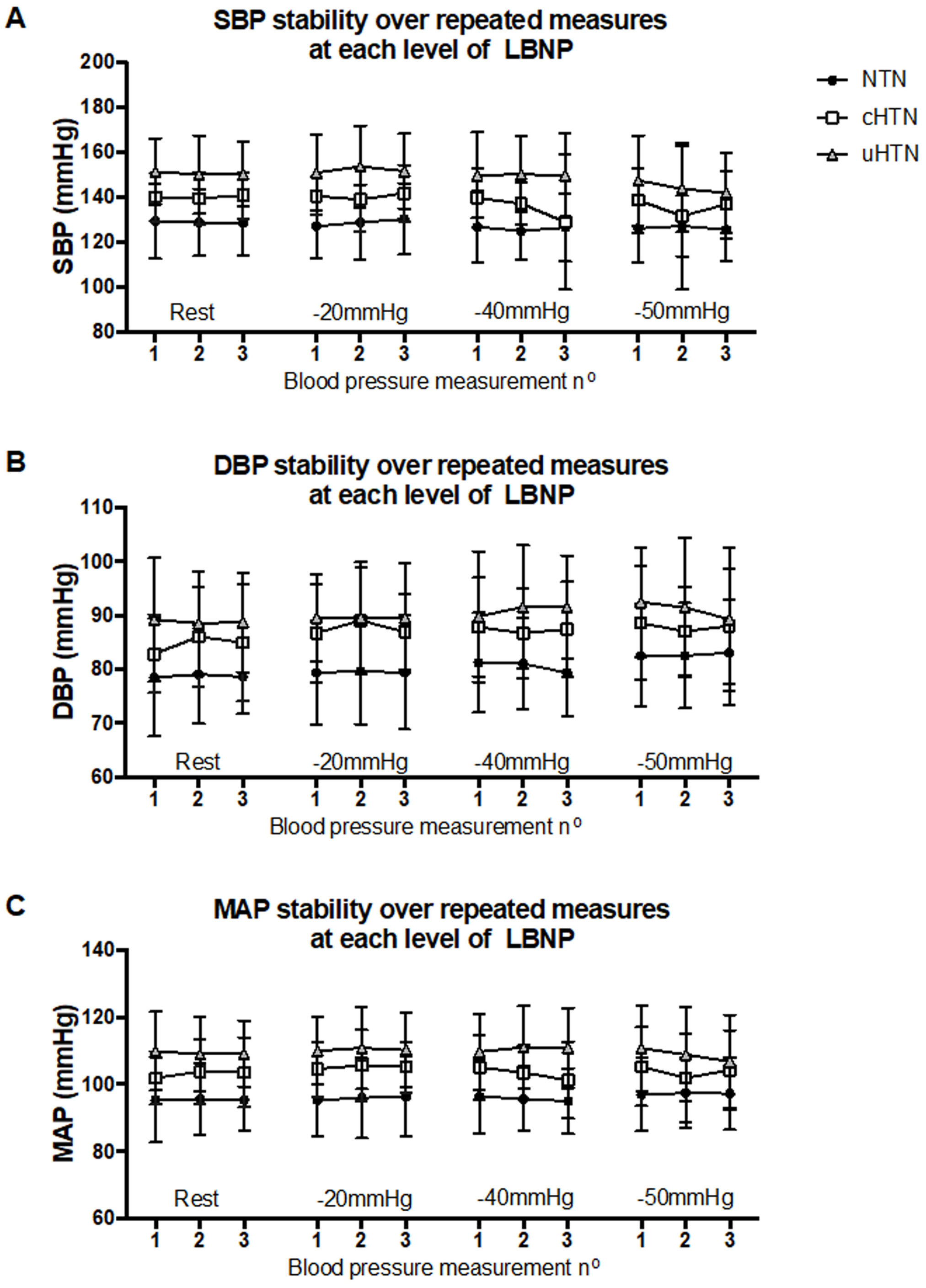

Figure S4: Blood pressure stability for **A)** Systolic blood pressure, **B)** Diastolic blood and, **C)** Mean arterial blood pressure stability over each ~5minute LBNP period. A total of 3 measures were taken at each level across the 5 minutes, spread at approximately 1.5minutes, Data are shown as mean for each group ± standard deviation.

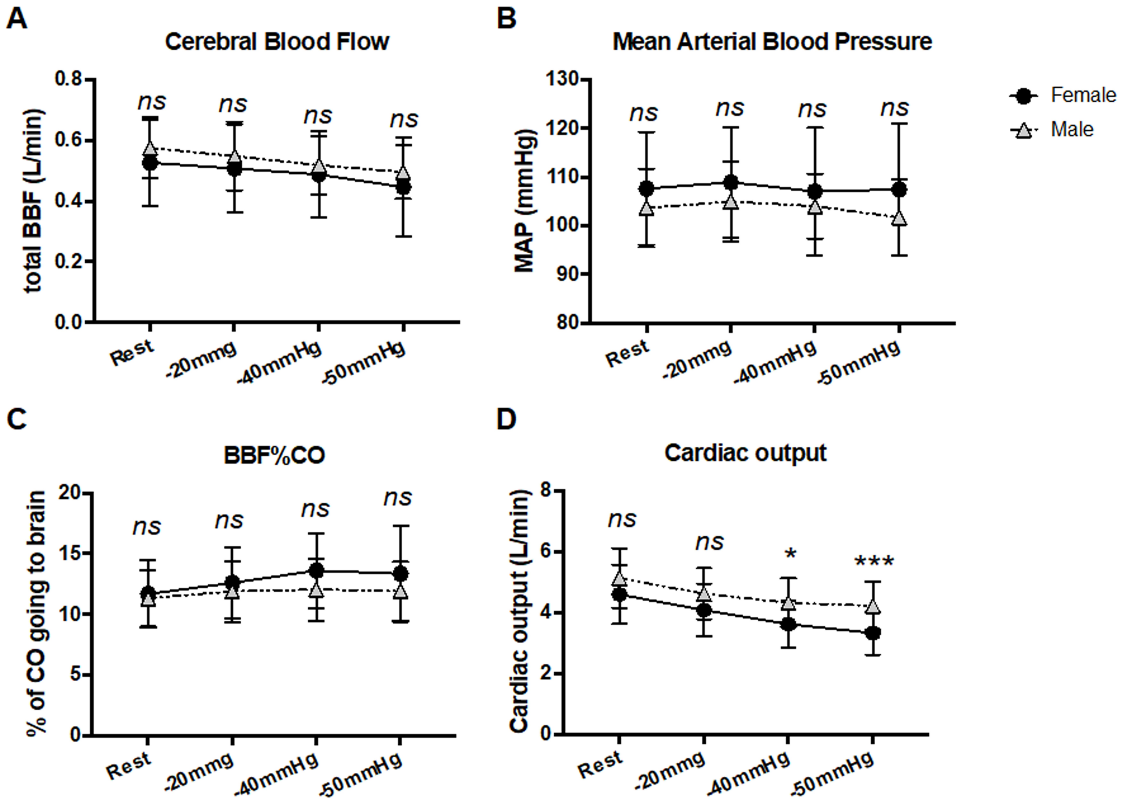

Figure S5: LBNP data analysed by sex, A) total cerebral blood flow (L/min), **B)** Mean arterial pressure (mmHg), **C)** Percentage of the cardiac output going to the brain, **D)** Cardiac output (L/min). Data shown are mean +/- standard deviation.
