# Supplementary figures and images for: Cerebral Blood Flow Response to Simulated Hypovolemia in Essential Hypertension: A Magnetic Resonance Imaging Study
Source: Hypertension. 2019 Oct 28;74(6):1391–8. doi: 10.1161/HYPERTENSIONAHA.119.13229 (PMC7069391; doi:10.1161/HYPERTENSIONAHA.119.13229)

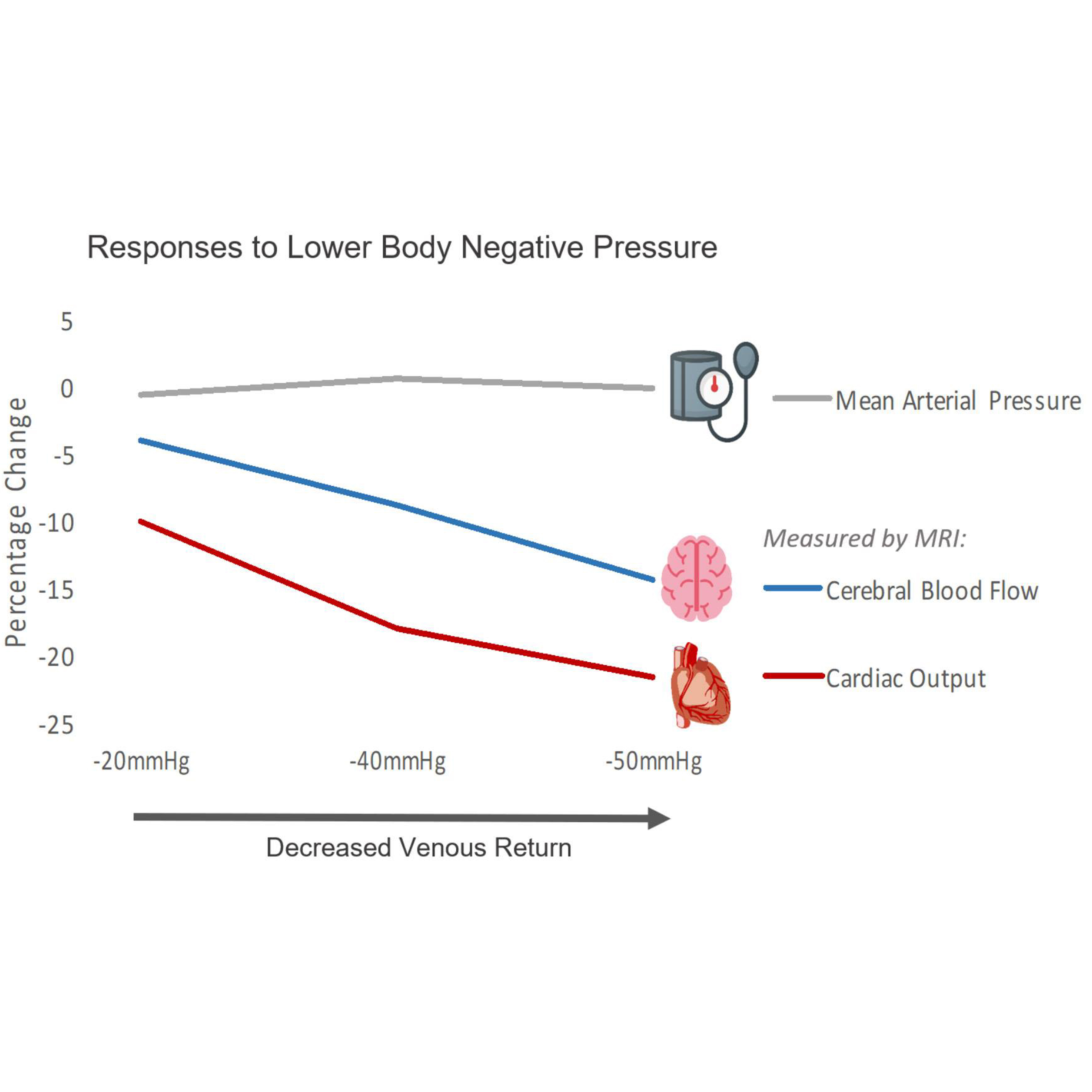

Supplement: Supplementary file 2 [file hyp-74-1391-s002.jpg]
