# Supplementary material for: Cerebral Blood Flow Response to Simulated Hypovolemia in Essential Hypertension: A Magnetic Resonance Imaging Study
Source: Hypertension. 2019 Oct 28;74(6):1391–8. doi: 10.1161/HYPERTENSIONAHA.119.13229 (PMC7069391; doi:10.1161/HYPERTENSIONAHA.119.13229)
Supplement: Supplementary file 3 [file hyp-74-1391-s003.pdf]

Change of Authorship Form
(Must be completed and signed by ALL authors)

Please check all that apply
__X__ New author(s) have been added.
_____ There is a change in the order of authorship.
_____ An author wishes to remove his/her name. An author's name may only be removed at his/her own request and a letter signed by the author should accompany this form.

Manuscript Number HYPE201913229-R1
Manuscript Title Cerebral blood flow response to simulated hypovolaemia in essential hypertension: an MRI study

FORMER AUTHORSHIP
Please list **ALL AUTHORS** in the same order as the original submission. For more than 12 use an extra sheet.

**Print Name**		**Print Name**	
Name (1)	Sandra Neumann	Name (7)	Zoe H Adams
Name (2)	Amy E Burchell	Name (8)	Jonathan CW Brooks
Name (3)	Christopher B Lawton	Name (9)	Angus K Nightingale
Name (4)	Daniel Burden	Name (10)	Julian FR Paton
Name (5)	Melissa Underhill	Name (11)	Mark CK Hamilton
Name (6)	Matthew D Kobetic	Name (12)	Emma C Hart

NEW AUTHORSHIP-All authors must sign below agreeing to the new changes in authorship. The authorship order must match the new title page of the manuscript.

Name (1)	Sandra Neumann	Signature	Sandra Berlau Neumann	Date	01/08/19
Name (2)	Amy E Burchell	Signature	Amy Burchell	Date	01/08/19
Name (3)	Jonathan CL Rodrigues	Signature	Jon Rodrigues	Date	01/08/19
Name (4)	Christopher B Lawton	Signature	Christopher Lawton	Date	01/08/19
Name (5)	Daniel Burden	Signature	Dan Burden	Date	01/08/19
Name (6)	Melissa Underhill	Signature	Melissa Underhill	Date	01/08/19
Name (7)	Matthew D Kobetic	Signature	Matt Kobetic	Date	01/08/19
Name (8)	Zoe H Adams	Signature	Zoe Adams	Date	01/08/19
Name (9)	Jonathan CW Brooks	Signature	Jon Brooks	Date	01/08/19
Name (10)	Angus K Nightingale	Signature	Angus Nightingale	Date	01/08/19
Name (11)	Julian FR Paton	Signature	Julian Paton	Date	01/08/19
Name (12)	Mark CK Hamilton	Signature	Mark Hamilton	Date	01/08/19
Name (13)	Emma C Hart	Signature	Emma Hart	Date	01/08/19

Please fax to 214-706-1565 or email hypertension@heart.org.
